# Treatment patterns among patients with moderate-to-severe ulcerative colitis in the United States and Europe

**DOI:** 10.1371/journal.pone.0227914

**Published:** 2020-01-16

**Authors:** Alessandro Armuzzi, Marco daCosta DiBonaventura, Miriam Tarallo, James Lucas, Daniel Bluff, Benjamin Hoskin, Danielle Bargo, Joseph C. Cappelleri, Daniel Quirk, Leonardo Salese

**Affiliations:** 1 IBD Unit, Presidio Columbus Fondazione Policlinico A. Gemelli IRCCS–Università Cattolica del Sacro Cuore, Rome, Italy; 2 Patient Health Impact, Pfizer Inc, New York, NY, United States of America; 3 Patient Health Impact, Pfizer Inc, Rome, Italy; 4 Adelphi Real World, Macclesfield, SK, United Kingdom; 5 Biostatistics, Pfizer Inc, Groton, CT, United States of America; 6 Medical Affairs, Pfizer Inc, Collegeville, PA, United States of America; Humanitas University, ITALY

## Abstract

**Objective:**

The aim of the present study is to examine how moderate-to-severe ulcerative colitis (UC) is currently managed in real-world clinical practice across the United States (US) and European Union Five (EU5; France, Germany, Italy, Spain, and the United Kingdom).

**Methods:**

Data from the 2017 Adelphi Inflammatory Bowel-Disease Specific Programme (IBD-DSP) were used. The IBD-DSP is a database of patient chart information abstracted by selected gastroenterologists across the US and EU5. Eligible gastroenterologists who agreed to participate were asked to complete patient record forms for the next seven consecutive eligible adult patients with UC. Only charts from patients with moderate-to-severe UC were included in the analysis (defined as those with documented administration of either an immunosuppressant [IM] or a biologic). Treatment patterns were reported descriptively.

**Results:**

411 and 1191 patient charts were included in the US and EU5 (mean ages 44.2 and 39.6 years; 53.0% and 43.5% female), respectively. For those with complete treatment history, 40.7% and 52.9% used either an IM or biologic as their first treatment (with or without steroids). Usage of these therapies increased in subsequent lines. The percentage of patients treated with combination therapy (*i*.*e*., biologic therapy with a concomitant IM) in first line generally varied between 10–20% (*e*.*g*., US: adalimumab (ADA), 10.8%; infliximab (IFX), 18.2%; EU5: ADA, 12.5%; IFX, 19.9%), though increased in later lines in the EU5. Among patients currently using a biologic therapy, between 10–40% of patients used a higher than indicated dose or greater than indicated dosing frequency during maintenance (*e*.*g*., US: IFX, 37.1%; ADA, 13.4%; EU5: IFX, 39.1%; ADA, 36.1%). In both the US and EU5, the primary reason for switching therapy was efficacy-related.

**Conclusions:**

In this analysis, many patients with moderate-to-severe UC use an IM or biologic as their first therapy after diagnosis. Combination therapy and dose escalation are also common, and underscore the challenges with managing this patient population.

## Introduction

Ulcerative colitis (UC) is an idiopathic, chronic inflammatory disease of the colon that is characterized by intermittent periods of disease flaring and remission [1, 2]. UC affects approximately 900,000 people in the United States (US), 1.5 million people in Europe, and over 3 million people worldwide [3–5]. Although the primary clinical symptoms include rectal bleeding, diarrhea, urgency, and tenesmus, patients can also experience a range of additional symptoms such as abdominal pain and fatigue [2, 6–8].

The primary therapeutic goal in UC is to induce and maintain long-term disease remission [8]; however, there is no single treatment pathway for patients. A number of guidelines (*e*.*g*., from the American College of Gastroenterology [8], the European Crohn’s and Colitis Organization [9], and the Toronto Consensus [10]) have been published to review the most current treatment options and the corresponding supportive evidence for each. These guidelines recommend the use of either conventional therapies (*i*.*e*., aminosalicylates [5-ASAs], corticosteroids, immunosuppressants [IMs]), biologic therapies (*i*.*e*., infliximab [Biogen Similars; Janssen Biotech; Merck & Co.; Napp Pharmaceuticals; Pfizer Inc; Sandoz], adalimumab [Amgen; AbbVie; Biogen Similars; Boehringer Ingelheim; Merck & Co.; Mylan; Sandoz], golimumab [Janssen Biotech; Merck & Co.], vedolizumab [Takeda Pharmaceuticals]) and/or small molecule JAK inhibitor (i.e. tofactinib [Pfizer Inc]) to achieve clinical goals depending on severity of disease.

However, it remains unclear how moderate-to-severe UC is currently managed in real-world clinical practice. A few studies have examined the treatment patterns of UC, though they have often relied upon administrative claims databases and were conducted prior to the approval of several of the available treatments. For example, an article by Loftus and colleagues (2014) examined treatment patterns among patients who initiated IM therapy but, by definition, did not include patients who were treated by biologic therapy [11]. Conversely, an article by Patel and colleagues (2017) focused exclusively on patients who newly initiated biologic therapy [12]. Both articles, as well as a study by Rubin and colleagues (2014), were conducted prior to the approval of tofacitinib and vedolizumab (and, in some cases, prior to the approval of adalimumab and golimumab as well) [13].

The goal of the present study was to use medical record data and examine how patients with moderate-to-severe UC are currently treated across clinical practices in the US and Europe. Specifically, the objectives of this study were to assess the treatments used by sequential line of therapy, the frequency of IMs being used in combination with biologic therapy, the frequency of dose escalation with biologic therapies, and the major reasons for treatment switching.

## Methods

### Data sources

Data from the 2017 US and European Union Five (EU5; France, Germany, Italy, Spain, and the United Kingdom) Inflammatory Bowel Disease (IBD)-Disease Specific Programme (DSP) were used. The DSP data consists of medical chart information abstracted by each patient’s physician. The methods of the DSP have been previously published [14], though are summarized briefly below.

To acquire these data for the IBD-DSP, gastroenterologists in the US and EU5 were recruited by phone to participate in the study. Potential physician respondents were identified from publically available lists of healthcare professionals. Field-based interviews were then conducted to ensure eligibility (*i*.*e*., gastroenterologists had to be board-certified, have been a qualified physician for between four and 40 years, make treatment decisions for more than eight patients with Crohn’s disease and seven patients with UC per month, and be active in the treatment management of their patients). Eligible gastroenterologists who agreed to participate in the IBD-DSP were then asked to complete patient record forms for the next seven consecutive eligible patients with UC. Patients were considered eligible if they were adults with UC and had a history of moderate-to-severe disease. More specifically, patients were eligible to be included in the IBD-DSP if they were 18 years or older, had a diagnosis of UC, had received either a steroid, IM, or biologic for their UC, had been considered moderate or severe at some point based on the physician’s perception, or had a full Mayo score of >4 at some point. The patient record form was completed using an electronic data collection platform and included questions on the patient’s demographics, clinical state, current treatment, and general patient management.

The data collection forms were piloted with physicians prior to study implementation to ensure sufficient content validity [14]. The data were fully anonymized prior to analysis, and patients provided written informed consent for data from their medical records to be used. The protocol and study materials were reviewed and approved by the Western Institutional Review Board (Puyallup, WA, USA).

### Sample

From the UC patient charts that are present in the IBD-DSP database, our present study only included those who had prior exposure to either an IM or biologic.

### Measures

#### Patient demographics and health history

The patients’ age at data capture, gender, ethnic origin (as available/allowable in each country), employment status, height and weight (to calculate body mass index), smoking status, and disease duration were all abstracted from the medical charts.

#### Treatment history

The current treatments prescribed, their method of administration, dose and frequency, and duration of treatment to date were all abstracted. Complete treatment history (*i*.*e*., prior treatments along with the reason(s) for switching) was also reported.

### Statistical analysis

Analyses were conducted separately by region (US vs. EU5) and were largely descriptive, reporting counts and frequencies for categorical variables and means and standard deviations for continuous variables.

## Results

### Sample characteristics

A total of 411 patient charts from the US were included in the analyses; 53.0% of the patients were female and the mean age was 44.2 years (standard deviation [SD] = 14.2). The mean duration since diagnosis was 4.5 years (SD = 5.0) (Table 1). Across the EU5, 1191 patient charts were included (France: N = 331, Germany: N = 271, Italy: N = 207, Spain: N = 250, United Kingdom: N = 132). Compared with US patients, EU5 patients were slightly younger (mean age = 39.6, SD = 13.7), were more likely to be male (56.5%), and were diagnosed for longer (mean = 4.9 years, SD = 5.9 years).

**Table 1:** Demographics of the study sample.

	US N = 411	EU5 N = 1191
Country, n (%)		
	France	--	331 (27.8)
Germany	--	271 (22.8)
Italy	--	207 (17.4)
United Kingdom	--	132 (11.1)
Spain	--	250 (21.0)
US	411 (100.0)	
Age (years), mean (SD)	44.2 (14.2)	39.6 (13.7)
Sex, n (%)		
Male	193 (47.0)	673 (56.5)
Female	218 (53.0)	518 (43.5)
Race/ethnicity, n (%)		
White	315 (76.6)	1065 (89.4)
Non-white	96 (23.4)	126 (10.6)
Employment, n (%)^a^		
Working full time	246 (63.7)	654 (56.5)
Working part time	45 (11.7)	108 (9.3)
On long-term sick leave	2 (0.5)	32 (2.8)
Homemaker	42 (10.9)	80 (6.9)
Student	12 (3.1)	118 (10.2)
Retired	35 (9.1)	91 (7.9)
Unemployed	4 (1.0)	75 (6.5)
Body mass index, mean (SD)	26.2 (4.1)	23.7 (3.5)
Smoking status, n (%)^b^		
Current smoker	21 (5.6)	180 (16.3)
Former smoker	98 (26.3)	333 (30.2)
Never smoked	253 (68.0)	591 (53.5)
Duration since diagnosis (years), mean (SD)	4.5 (5.0)	4.9 (5.9)
Disease extent, n (%)		
Proctitis	68 (16.5)	151 (12.7)
Proctosigmoiditis	74 (18.0)	218 (18.3)
Left-sided	112 (27.3)	374 (31.4)
Pancolitis	139 (33.8)	416 (34.9)

^a^Note employment data were only available for N = 386 and N = 1158 of US and EU5 patient charts, respectively. Percentages exclude these missing data

^b^Note smoking history data were only available for N = 372 and N = 1104 of US and EU5 patient charts, respectively. Percentages exclude these missing data

EU5, European Union Five (France, Germany, Italy, Spain, and the United Kingdom); SD, standard deviation; US, United States.

### Overall treatment patterns

**Fig 1:**
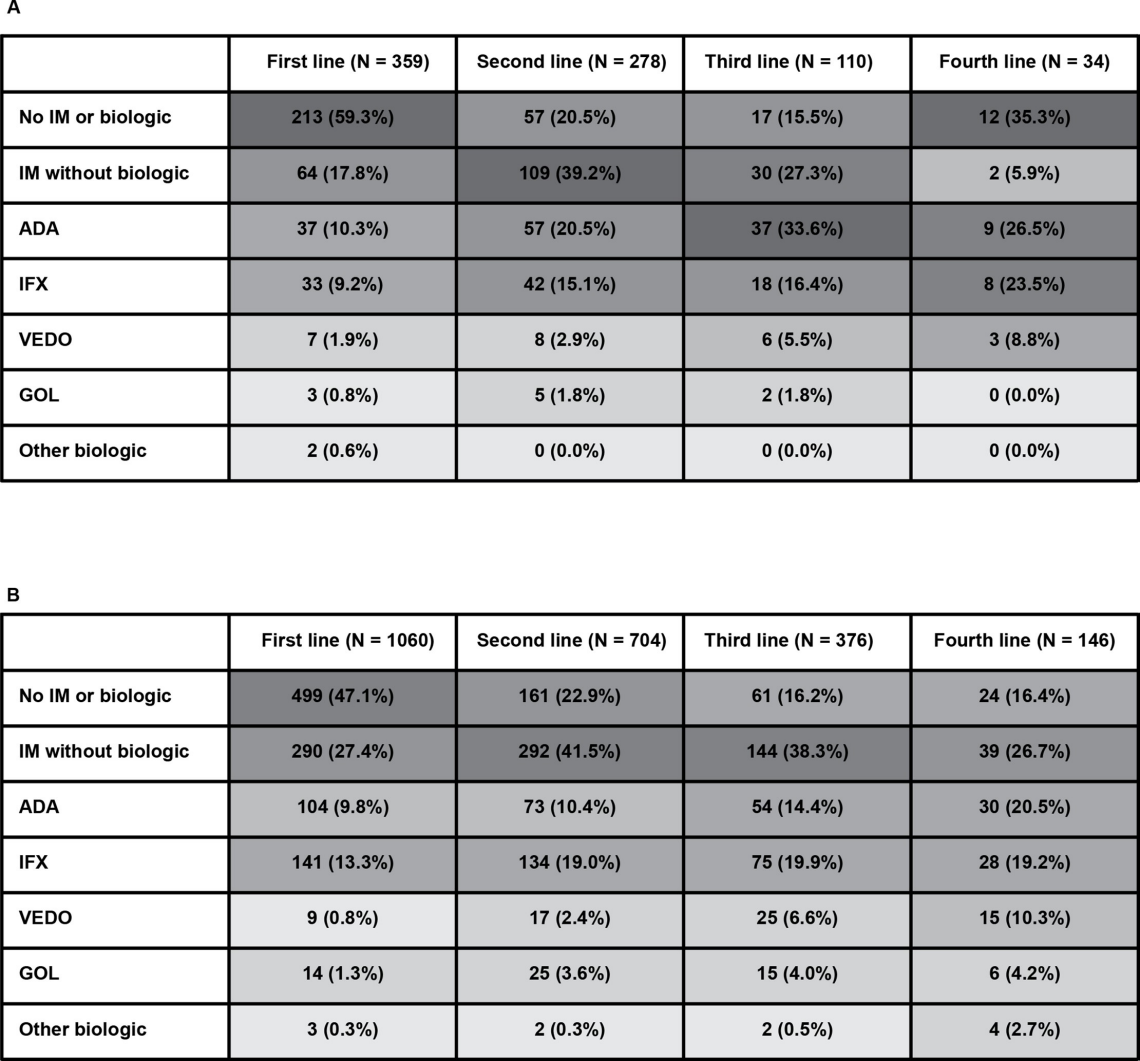
Heatmap of treatment usage by line of therapy. (A) Patients in the US. (B) Patients in the EU5 Cells are shaded in direct proportion to their associated percentages, with darker colors representing higher percentages. Column sample sizes represent the number of patients who proceeded to that line of therapy. For example, all patients (N = 359 and N = 1060 for the US and EU5, respectively) had a first-line treatment though only a proportion of those switched to a subsequent treatment (*i*.*e*., second line) ADA, adalimumab; EU5, European Union Five (France, Germany, Italy, Spain, and the United Kingdom); GOL, golimumab; IFX, infliximab; IM, immunosuppressant; US, United States; VEDO, vedolizumab.

To assess the full chronology of the treatments used, only patient charts with a complete treatment history (*i*.*e*., the documentation of all treatments used since diagnosis) were included in the overall treatment patterns analysis (US: N = 359, EU5: N = 1060). Treatments used by line of therapy are reported in Fig 1 for both the US and EU5.

In the US, most patients (59.3%) began treatment with 5-ASAs and/or steroids, as their first-line treatment (Fig 1A). However, the remaining 40.7% used either an IM or biologic (with or without steroids) as their first treatment. Use of IM therapy (without a biologic) was higher in the second line (39.2%) and third line (27.3%) compared with the first line (17.8%). Similarly, the use of biologic therapy also increased in the second line (overall = 40.3%; adalimumab [ADA] = 20.5%; infliximab [IFX] = 15.1%; vedolizumab [VEDO] = 2.9%; golimumab [GOL] = 1.8%) and again in the third line (overall = 57.3%; ADA = 33.6%; IFX = 16.4%; VEDO = 5.5%; GOL = 1.8%).

The pattern was similar in the EU5 (Fig 1B). Forty-seven percent (47.1%) used 5-ASAs and/or steroids in the first line while the remaining 52.9% used either an IM without a biologic (27.4%) or a biologic (25.6%; mostly either IFX or ADA). The usage of an IM without a biologic was higher in both the second (41.5%) and third (38.3%) lines compared with the first line (27.4%). Similarly, the use of biologic therapy became increasingly common in subsequent lines (*e*.*g*., 35.7% in the second line and 45.5% in the third line).

IMs were often administered concomitantly with biologic therapy in the US (*e*.*g*., first line: ADA = 10.8%; IFX = 18.2%; VEDO = 42.9%) (Table 2).

**Table 2:** Frequency of combination therapy among patients with moderate-to-severe UC in the US.

Treatment, n (%)	First line (N = 359)		Second line (N = 278)		Third line (N = 110)		Fourth line (N = 34)	
**ADA**								
Without IM	33	(9.2)	55	(19.8)	32	(29.1)	6	(17.6)
With IM	4	(1.1)	2	(0.7)	5	(4.5)	3	(8.8)
% combination therapy		**10.8**	** **	**3.5**	** **	**13.5**	** **	**33.3**
**IFX**								
Without IM	27	(7.5)	30	(10.8)	15	(13.6)	7	(20.6)
With IM	6	(1.7)	12	(4.3)	3	(2.7)	1	(2.9)
% combination therapy		**18.2**	** **	**28.6**	** **	**16.7**	** **	**12.5**
**VEDO**								
Without IM	4	(1.1)	7	(2.5)	5	(4.5)	2	(5.9)
With IM	3	(0.8)	1	(0.4)	1	(0.9)	1	(2.9)
% combination therapy		**42.9**	** **	**12.5**	** **	**16.7**	** **	**33.3**
**GOL**								
Without IM	3	(0.8)	4	(1.4)	2	(1.8)	0	(0.0)
With IM	0	(0.0)	1	(0.4)	0	(0.0)	0	(0.0)
% combination therapy		**0.0**	** **	**20.0**	** **	**0.0**	** **	**0.0**
**Any of the above**								
Without IM	67	(18.7)	96	(34.5)	54	(49.1)	15	(44.1)
With IM	13	(3.6)	16	(5.8)	9	(8.2)	5	(14.7)
% combination therapy		**16.3**	** **	**14.3**	** **	**14.3**	** **	**20.0**

ADA, adalimumab; GOL, golimumab; IFX, infliximab; IM, immunosuppressant; UC, ulcerative colitis; US, United States; VEDO, vedolizumab.

The frequency of combination therapy did not appreciably differ across lines of therapy, though small samples limited the extent to which this could be examined. In the EU5, combination therapy was also common (Table 3).

**Table 3:** Frequency of combination therapy among patients with moderate-to-severe UC in the EU5.

Treatment, n (%)	First line (N = 1060)		Second line (N = 704)		Third line (N = 376)		Fourth line (N = 146)	
**ADA**								
Without IM	91	(8.6)	56	(8.0)	40	(10.6)	21	(14.4)
With IM	13	(1.2)	17	(2.4)	14	(3.7)	9	(6.2)
% combination therapy		**12.5**		**23.3**		**25.9**		**30.0**
**IFX**								
Without IM	113	(10.7)	95	(13.5)	51	(13.6)	18	(12.3)
With IM	28	(2.6)	39	(5.5)	24	(6.4)	10	(6.8)
% combination therapy		**19.9**		**29.1**		**32.0**		**35.7**
**VEDO**								
Without IM	8	(0.8)	11	(1.6)	20	(5.3)	13	(8.9)
With IM	1	(0.1)	6	(0.9)	5	(1.3)	2	(1.4)
% combination therapy		**11.1**		**35.3**		**20.0**		**13.3**
**GOL**								
Without IM	13	(1.2)	22	(3.1)	12	(3.2)	4	(2.7)
With IM	1	(0.1)	3	(0.4)	3	(0.8)	2	(1.4)
% combination therapy		**7.1**		**12.0**		**20.0**		**33.3**
**Any of the above**								
Without IM	225	(21.2)	184	(26.1)	123	(32.7)	56	(38.4)
With IM	43	(4.1)	65	(9.2)	46	(12.2)	23	(15.8)
% combination therapy		**16.0**		**26.1**		**27.2**		**29.1**

ADA, adalimumab; EU5, European Union Five (France, Germany, Italy, Spain, and the United Kingdom); GOL, golimumab; IFX, infliximab; IM, immunosuppressant; UC, ulcerative colitis; VEDO, vedolizumab

A total of 19.9% of patients using IFX also used an IM in the first line; 12.5%, 11.1%, and 7.1% of patients using ADA, VEDO, and GOL, respectively, were also using an IM. Combination therapy was more common in the second (26.1%) and later lines (third line: 27.2%, fourth line: 29.1%) compared with the first line (16.0%) across any of ADA, IFX, VEDO, and GOL.

In the US, duration of treatment was longest in the second and first lines of treatment (65.1 and 63.4 weeks, respectively), followed by the fourth and third lines (43.5 and 42.9 weeks, respectively). The duration of treatment was similar across the first three lines of treatment in the EU5 group, and slightly higher in the fourth line: 55.6 weeks (first line), 56.3 weeks (second line), 56.7 weeks (third line), and 60.1 weeks (fourth line).

### Dose escalation

**Fig 2:**
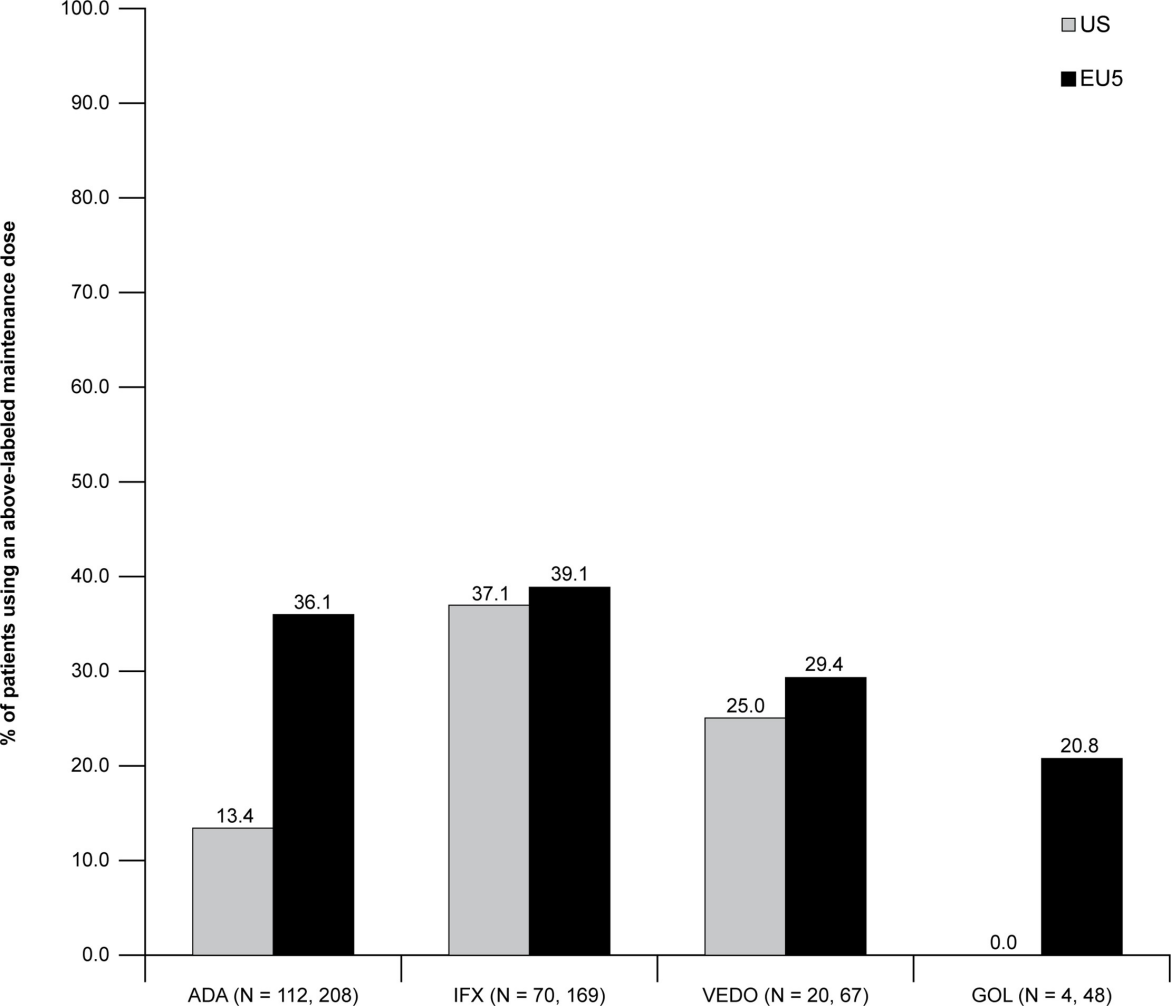
Frequency of dose escalation during maintenance for patients in the US and EU5. Sample sizes shown in the x-axis refer to the US and EU5, respectively. ADA, adalimumab; EU5, European Union Five (France, Germany, Italy, Spain, and the United Kingdom); GOL, golimumab; IFX, infliximab; US, United States; VEDO, vedolizumab.

Among patients currently using a biologic for maintenance therapy (and who had been in maintenance for >3 months), physicians indicated the current dose and frequency prescribed. This was compared against the approved label for each treatment and used to calculate the proportion of patients using an escalated dose (*i*.*e*., a higher-than-indicated dose or greater-than-indicated dosing frequency). These results are reported in Fig 2. Across both regions, nearly 40% of patients on IFX (37.1% and 39.1% in the US and EU5, respectively) were using an escalated dose. In the EU5, 20.8–36.1% of patients using the remaining biologic treatments were using an escalated dose. These rates were lower in the US (ADA = 13.4%, VEDO = 25.0%, and GOL = 0.0%), though small sample sizes for VEDO and GOL (N = 20 and 4, respectively) limited the ability to examine dose escalation of these treatments.

### Reasons for switching

For patients who used more than one therapy, the reasons for switching from their previous therapy to their current therapy were recorded. As switching decisions are often complex, physicians could identify multiple reasons for each switch. The data were organized into higher-level categories for analysis and are presented by line of therapy as shown in Tables 4 and 5 (*e*.*g*., data are shown separately for patients who switched from their first to second therapy compared with patients who switched from their second to their third).

**Table 4:** Reasons for switching from previous treatment to current treatment among patients in the US.

	1^st^ to 2^nd^ treatment (N = 165)		2^nd^ to 3^rd^ treatment (N = 72)		3^rd^ to 4^th^ treatment (N = 17)	
Efficacy, n (%)	159	(96.4)	70	(97.2)	14	(82.4)
Safety/tolerability, n (%)	23	(13.9)	8	(11.1)	4	(23.5)
Mode of administration/ convenience, n (%)	59	(35.8)	35	(48.6)	6	(35.3)
Financial reasons, n (%)	9	(5.5)	0	(0.0)	0	(0.0)
Biosimilar switch, n (%)	1	(0.6)	1	(1.4)	0	(0.0)
Other, n (%)	5	(3.0)	2	(2.8)	0	(0.0)

Note that reasons for switching are only reported based on the change from the prior treatment to the current treatment (*e*.*g*., a patient on their second therapy will be represented in the 1^st^ to 2^nd^ treatment column). Patients on their first therapy (N = 81) were excluded as they have not switched. Physicians could select more than one reason for the switch

US, United States.

**Table 5:** Reasons for switching from previous treatment to current treatment among patients in the EU5.

	1^st^ to 2^nd^ treatment (N = 323)		2^nd^ to 3^rd^ treatment (N = 221)		3^rd^ to 4^th^ treatment (N = 82)	
Efficacy, n (%)	311	(96.3)	197	(89.1)	67	(81.7)
Safety/tolerability, n (%)	83	(25.7)	59	(26.7)	16	(19.5)
Mode of administration/ convenience, n (%)	71	(22.0)	30	(13.6)	12	(14.6)
Financial reasons, n (%)	8	(2.5)	6	(2.7)	3	(3.7)
Biosimilar switch, n (%)	14	(4.3)	11	(5.0)	3	(3.7)
Other, n (%)	14	(4.3)	7	(3.2)	7	(8.5)

Note that reasons for switching are only reported based on the change from the prior treatment to the current treatment (*e*.*g*., a patient on their second therapy will be represented in the 1^st^ to 2^nd^ treatment column). Patients on their first therapy (N = 356) were excluded as they have not switched. Physicians could select more than one reason for the switch

EU5, European Union Five (France, Germany, Italy, Spain, and the United Kingdom).

In the US, the primary reason for switching therapy (cited in 96.4%, 97.2%, and 82.4% of switches in the first, second, and third line, respectively) was due to an efficacy-related reason (*e*.*g*., “initial non-response”, “remission not maintained”, “lack of alleviation of pain”) (Table 4). However, mode of administration/convenience-related reasons were the next most frequently observed (cited in 35.3–48.6% of switches, depending upon line). These reasons included things such as “I wanted to use an advanced therapy that can be used as a monotherapy” or “frequency of injections”.

Similar results were observed in the EU5 (Table 5). Efficacy-related reasons were the most frequently cited (96.3% when switching from their first-line therapy to 81.7% when switching from their third-line therapy). However, unlike the US, safety-related reasons (*e*.*g*., “lack of tolerability”, “side effects”) were the next most common reasons for switching followed closely by mode of administration/convenience.

## Discussion

It is important to assess the real-world clinical practice of therapies for UC. This study provides an updated perspective on the treatment selections, use of combination therapy, dosages used, and reasons for switching among patients with moderate-to-severe UC. Contrary to past studies that have relied upon administrative claims data [11–13], our study’s use of medical record data offers a couple of advantages. First, medical records have the ability to provide a longitudinal view of the treatment pathway as administrative claims (particularly in the US) may have difficulty following patients as they switch health insurance plans and may favor patients with certain types of insurance. Second, medical record data can also help inform reasons for switching treatments, which is difficult to acquire elsewhere.

Our results suggest immunosuppressants and biologics are initiated early on in the disease course for many patients. Patients who become moderate to severe (as they may not all have been moderate to severe at diagnosis) used either an IM (approximately 18%) or biologic (approximately 23%) (with or without steroids) as their very first treatment. This finding underscores the difficulty in managing these patients as many present with such severe disease that conventional therapies alone are not considered an adequate initial therapy. Further, it may also illustrate the unmet need, as with so few treatment options available, physicians may opt to be aggressive to maximize the time prior to colectomy.

As expected, the use of biologic therapy increases dramatically in subsequent lines. Specifically, the use of ADA and IFX were highest, followed by VEDO (which was used disproportionately in later lines). The use of GOL was modest in both the US and EU5, as was the use of unapproved biologics.

This study also explored the frequency of combination therapy. The use of an IM along with a biologic is frequently used to inhibit the development of anti-drug antibodies and maximize the efficacy of the biologic treatment [15]. For example, the UC-SUCCESS trial demonstrated significantly better efficacy of combination therapy (azathioprine plus IFX) compared with monotherapy over a 16-week period [16]. Our study suggests that between 10–30% of patients use combination therapy, depending upon the specific biologic used (and, potentially, the line of therapy). This is slightly lower than rates reported from a recent US administrative claims database [17]. However, the data collection form encouraged physicians to consider any add-on of therapy to be a new line so if the IM was not administered concomitantly with the biologic therapy then the database would not consider this combination therapy. Therefore, these results may underestimate the frequency of combination therapy depending upon the timing in which IMs were administered. Additional research would be necessary.

Another important aspect to the management of patients using biologic therapy is that of dose escalation or the practice of using a higher-than-labeled dose or more-frequent-than-labeled administration. As noted by therapeutic drug-monitoring guidelines [15], the use of dose escalation may be warranted to maximize efficacy. The present study found that between 10–40% of patients, depending upon the specific biologic used, increased the dose and/or the frequency of administration during maintenance. This is generally consistent with studies in the US using administration claims data, which reported rates between 24–39% for ADA, IFX, and VEDO [17]. Rubin and colleagues 2017 reported slightly lower rates (13% and 29% for ADA and IFX, respectively) though they used a more restrictive definition; patients needed to have used double the daily dose for this to be considered an escalation [13]. Regardless, our results underscore the challenge in managing these patients, as a significant percentage require a higher-than-indicated dose to maintain (or regain) efficacy.

Physicians also provided direct insight into their reasons for switching therapies. Reasons for switching is multifactorial but almost universally included some aspect of efficacy. Interestingly, in the US, mode of administration/convenience concerns featured prominently as reasons for switching, even more so than safety-related reasons. This suggests the importance of administrative burden and ease of dosing regimen when evaluating therapeutic options. The same was not necessarily true for patients in the EU5. Although mode of administration/convenience factors were still prominent, safety-related reasons were more frequently cited. Drug costs and other financial considerations did not frequently factor in to switching decisions for either region.

Although it may be tempting to draw comparisons between regions, it should be noted that the patient populations are different. For example, patients in the EU5 were younger, diagnosed for longer, and more likely to be male than patients in the US. As there is a lack of epidemiological evidence to suggest fundamental differences in the UC patient profile across these regions, these differences are likely attributed to sampling error (at either, or both, a physician and patient level). Any comparisons across regions must consider the demographic differences as well, which may explain variability in treatment patterns.

### Limitations

Several limitations should be noted. These data were abstracted from the medical chart without any auditing; any data entry errors or subjective interpretation of clinical data could have introduced additional measurement error.

Overall, the number of patients in the US group was relatively low, and the number of charts with complete treatment history was also modest across both groups. This influenced the degree to which comparisons can be made between groups, across treatment lines, and across treatments themselves. Additionally, the results were purely descriptive without any statistical adjustment for confounding variables.

A further limitation was the low numbers of patients reporting surgical intervention, and the lack of detail collected on the extent of the surgery. Further study would be needed to look at treatment patterns in relation to surgical interventions.

The very high rates of IM and/or biologic use as first line may represent the inclusion of patients with more severe disease at presentation. Additionally, there may be selection bias for the inclusion of gastroenterologists at academic centers who may be more inclined to treat aggressively at initial diagnosis. Furthermore, it is also possible that the physicians who were willing to participate may not represent the practice patterns at large. This, coupled with the low numbers of patients in the US group, means that it is not clear the extent to which the sample of patients represents the broader UC population. More research would be necessary.

## Conclusions

In this study of real-world management of patients with moderate-to-severe UC in the US and EU5, many patients received an IM or biologic treatment as their first therapy after diagnosis. Combination therapy with both IM and biologic therapy was also commonly reported. For those patients who used biologic therapy, between 10–40% received a higher-than-indicated dose and/or frequency. The most commonly cited reasons for treatment switching were efficacy-, convenience-, and safety-related. Collectively, these findings provide an updated perspective on the real-world clinical practice of therapies for UC and reinforce the challenges that still exist in managing patients with UC in both the US and Europe.
